# Electrical responses from human retinal cone pathways associate with a common genetic polymorphism implicated in myopia

**DOI:** 10.1073/pnas.2119675119

**Published:** 2022-05-20

**Authors:** Xiaofan Jiang, Zihe Xu, Talha Soorma, Ambreen Tariq, Taha Bhatti, Alexander J. Baneke, Nikolas Pontikos, Shaun M. Leo, Andrew R. Webster, Katie M. Williams, Christopher J. Hammond, Pirro G. Hysi, Omar A. Mahroo

**Affiliations:** ^a^Institute of Ophthalmology, University College London, London EC1V 9EL, United Kingdom;; ^b^Department of Ophthalmology, King’s College London, London SE1 7EH, United Kingdom;; ^c^Department of Twin Research and Genetic Epidemiology, King’s College London, London SE1 7EH, United Kingdom;; ^d^Medical Retina Service, Moorfields Eye Hospital, London EC1V 2PD, United Kingdom;; ^e^Inherited Eye Disease Service, Moorfields Eye Hospital, London EC1V 2PD, United Kingdom;; ^f^Physiology, Development and Neuroscience, University of Cambridge, Cambridge CB2 3EG, United Kingdom

**Keywords:** myopia, gap junctions, OFF signaling pathways, electroretinography, retinal cone photoreceptor cells

## Abstract

Myopia is the commonest visual impairment. Several genetic loci confer risk, but mechanisms by which they do this are unknown. Retinal signals drive eye growth, and myopia usually results from an excessively long eye. The common variant most strongly associated with myopia is near the *GJD2* gene, encoding connexin-36, which forms retinal gap junctions. Light-evoked responses of retinal neurons can be recorded noninvasively as the electroretinogram (ERG). We analyzed these responses from 186 adult twin volunteers who had been genotyped at this locus. Participants underwent detailed ERG recordings incorporating international standard stimuli as well as experimental protocols aiming to separate dark-adapted rod- and cone-driven responses. A mixed linear model was used to explore association between allelic dosage at the locus and international standard ERG parameters after adjustment for age, sex, and family structure. Significant associations were found for parameters of light-adapted, but not dark-adapted, responses. Further investigation of isolated rod- and cone-driven ERGs confirmed associations with cone-driven, but not rod-driven, a-wave amplitudes. Comparison with responses to similar experimental stimuli from a patient with a prior central retinal artery occlusion, and from two patients with selective loss of ON-bipolar cell signals, was consistent with the associated parameters being derived from signals from cone-driven OFF-bipolar cells. Analysis of single-cell transcriptome data revealed strongest *GJD2* expression in cone photoreceptors; bipolar cell expression appeared strongest in OFF-bipolar cells and weakest in rod-driven ON-bipolar cells. Our findings support a potential role for altered signaling in cone-driven OFF pathways in myopia development.

## Significance

Myopia prevalence has increased dramatically over recent decades. Genome-wide association studies have identified numerous loci, but mechanisms by which genotypic identity confers myopia susceptibility are unknown. The common variant most strongly associated with myopia is near a gene encoding retinal gap junctions. We analyzed retinal electrophysiological responses from 186 twins genotyped at this locus, finding association between cone-driven, but not rod-driven, electroretinogram signals and allelic genotype. Examination of responses to further, nonstandard testing protocols, together with recordings from patients with selective loss of bipolar cell signals, points to an effect on cone-driven hyperpolarizing (“OFF”) signals. The pattern of retinal expression of this gene appears consistent with these findings, which support a potential role for altered cone-driven signaling in myopia development.

Myopia is the commonest cause of visual impairment and can be associated with blinding complications. Prevalence has increased dramatically worldwide over recent decades (1). Myopia is highly heritable (2), and genome-wide association studies (GWASs) have identified numerous polymorphisms, each conferring some small susceptibility to myopia (3, 4). Although genetic changes cannot explain increasing prevalence over this time frame, genetic associations are useful in highlighting pathways potentially important in myopia development, which can inform future preventive strategies. Presently, as with GWAS findings in most diseases, mechanisms by which variants confer myopia susceptibility are not known.

The biggest contributor to myopia is the axial length of the eye; longer eyes lead to light from distant objects being focused in front of the retina. Eye growth is finely controlled across species. If a lens is placed permanently in front of a chick eye, the eye grows appropriately to neutralize the effect; this occurs even after optic nerve transection severs connection with the brain, indicating the process is driven locally (5). The retina somehow detects magnitude and direction of blur, and some studies have shown differences in retinal neuronal responses based on whether stimuli are myopically or hyperopically defocused (6–8). The retina directs growth of the sclera appropriately, which determines the eye’s axial length. Thus, GWAS loci that potentially relate to retinal signaling are of particular interest.

One locus consistently identified from GWAS investigations is rs524952 (3, 4, 9). Of the common myopia-linked polymorphisms, this has the strongest effect (estimated effect size of risk allele, −0.158 diopters of spherical equivalent) (9). This locus is near the gene *GJD2*, encoding connexin-36, which forms retinal gap junctions, permitting electrical coupling between several retinal neuronal types (10–12). Changes in patterns of expression of this protein might plausibly alter some characteristic of electrical signal transmission relevant to the drive for eye growth.

The electroretinogram (ERG) represents the summed electrical response of the retina to light stimuli and can be recorded noninvasively in vivo from human subjects. International standard stimulus protocols permit quantification of responses from rod and cone systems (13). Briefly, stimuli presented in the dark, following 20 min of dark adaptation, permit assessment of the rod system. Stimuli presented in the presence of a white background (that saturates the rod photoreceptors), following 10 min of light adaptation to the same background, enable assessment of the cone system. *SI Appendix*, Figs. S1 and S2 illustrate ERG recording and show the likely cellular origin of ERG components elicited by standard stimuli. ERG responses originate primarily from rod and cone photoreceptors, and from second-order neurons, namely depolarizing (“ON”) and hyperpolarizing (“OFF”) bipolar cells.

In this study, we sought to test the hypothesis that alterations in retinal electrophysiology might associate with allelic genotype at this myopia-susceptibility locus. Adult volunteers from the TwinsUK cohort (14) were recruited for detailed retinal recordings. ERGs were recorded from over 200 volunteers (15) in response to both standard and additional experimental protocols. The majority of participants were genotyped for allelic identity at this locus. The additional experimental protocols aimed to selectively interrogate dark-adapted rod and cone system responses.

To further clarify the neuronal origin of particular ERG components, we also analyzed responses to identical stimuli from patients in whom signals from particular retinal neurons had been lost. These included a patient with a prior central retinal artery occlusion (CRAO; removing both ON and OFF postphotoreceptoral signals) and two patients with complete congenital stationary night blindness (cCSNB; in whom signals from ON-bipolar cells are selectively lost). Interpreting the recordings from the healthy participants in light of findings in these patients leads us to associate the myopia-susceptibility locus with signals from cone-driven OFF-bipolar cells (possibly with the risk allele being associated with less hyperpolarization in these cells). We also consulted single-cell transcriptome studies to explore retinal *GJD2* expression, and these analyses were consistent with our findings.

## Results

### Demographics of Participants and Distribution of Risk Alleles.

In total, 210 participants were recruited for ERG recording. Genotypes and interpretable recordings were available from 186 subjects (95% female; mean [SD] age, 64.2 [9.7] y). The participant age and sex distribution reflects the wider demographics of the TwinsUK cohort; the registry was set up 30 y ago with the initial aim of investigating osteoporosis and osteoarthritis, conditions which are more prevalent in older females (14). Participants were grouped according to the number of risk alleles at the locus of interest. Thirty-seven participants had no risk alleles (designated “group 0”), 101 had one risk allele (“group 1”), and 48 had two risk alleles (“group 2”) at the locus of interest. The risk allele (associated with more myopic refractive error) and reference allele are A and T, respectively. Thus, group 0 was TT, and group 2 was AA, at this locus. There were no significant age differences between groups (*P* > 0.38). Mean (SD) axial lengths (available for 88% of participants) were 23.20 (1.38), 23.25 (1.06), and 23.38 (1.18) mm for groups 0, 1, and 2, respectively, with no significant difference between groups (*P* > 0.5 for pairwise comparisons). Mean (SD) spherical equivalent refractions (available for 95% of participants) were +0.36 (3.17), −0.03 (2.04), and −0.48 (2.31) diopters for groups 0, 1, and 2, respectively, with no significant difference between groups (*P* > 0.15 for pairwise comparisons). Most of the cohort did not have high refractive error: 82.5% had spherical equivalent refractions between −3 and +3 diopters. Median spherical equivalent refraction for the whole cohort was 0.25 diopters.

### ERG Responses to International Standard Dark-Adapted and Light-Adapted Protocols.

**Fig. 1.**
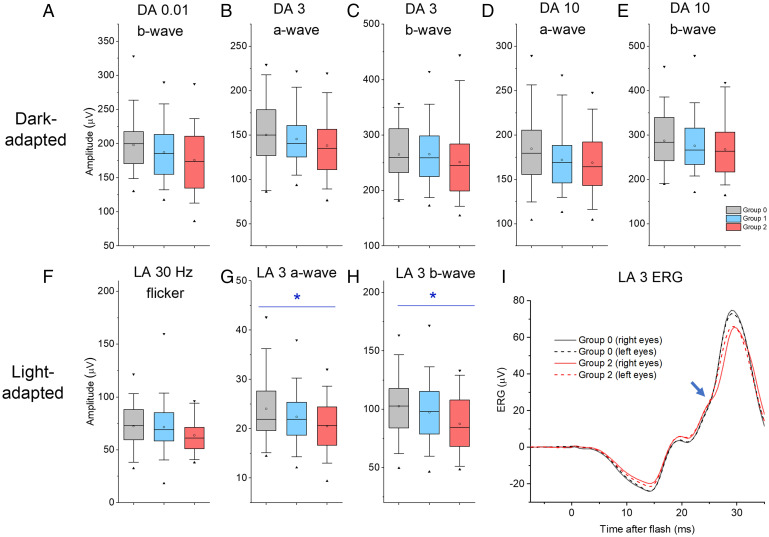
Amplitudes of ERG responses to international standard dark-adapted (DA) and light-adapted (LA) stimuli (flash intensities in cd⋅m^−2^⋅s). (*A*–*H*) Participants are grouped by number of myopia risk alleles at the locus of interest: Groups 0, 1, and 2 comprise participants with 0, 1, or 2 risk alleles, respectively (gray, blue, and red boxes). Maximal and minimal values are shown as filled black triangles; whiskers denote 5th and 95th centiles; boxes show median and upper and lower quartiles; and opencircles denote mean values. *A–E* show DA amplitudes and *F–H* show LA amplitudes. Asterisks denote significance (*G* and *H*). (*I*) Averaged light-adapted flash responses (LA 3 stimulus) for participants with no risk allele (group 0, black traces) and those homozygous for the risk allele (group 2, red traces). Solid and dashed traces show averages of right eye and left eye recordings, respectively. For group 0, the averaged a-waves and b-waves are larger compared with group 2. The blue arrow highlights an inflection or gradient change around 25 ms seen in group 2 traces (and not obviously present in group 0 traces).

Table 1 gives mean (SD) ERG amplitudes elicited by standard stimuli for each group. These are illustrated in Fig. 1. Table 1 also shows the results of mixed linear model associations with electrophysiological parameters as outcomes and allelic dosage at this locus as a predictor, adjusting for age, sex, and interindividual relatedness. None of the dark-adapted ERG amplitudes showed significant differences across groups, but both the light-adapted ERG a-wave and b-wave amplitudes showed significant associations. These are shown in Fig. 1 *G* and *H*. In multivariate analysis (*SI Appendix*, Table S1), association with the light-adapted a-wave amplitude remained significant (*P* = 0.046), and association with the light-adapted flicker ERG amplitude was also found to be significant (*P* = 0.046). No peak time parameters showed significant associations (although two of the light-adapted peak time parameters approached significance, with *P* values of 0.065 and 0.077 for flicker and b-wave peak times, respectively).

**Table 1.** Mean (SD) amplitudes for standard ERG parameters for participants grouped by number of risk alleles

Stimulus	Response component	Mean (SD) amplitudes, µV			*P* value (mixed model)
		No risk alleles (group 0), *n* = 37	One risk allele (group 1), *n* = 101	Two risk alleles (group 2), *n* = 48	
DA 0.01 (dim flash)	b-wave	197.7 (38.8)	187.1 (39)	175.6 (46.4)	0.078
DA 3 (standard flash)	a-wave	150.1 (35.2)	145.5 (28.9)	138.0 (34.1)	0.172
	b-wave	264.7 (50.6)	265.2 (52.5)	251.2 (67.4)	0.409
DA 10 (bright flash)	a-wave	184.4 (41)	171.9 (34.6)	168.7 (35.5)	0.054
	b-wave	286.7 (63)	275.5 (56.2)	267.3 (61.4)	0.143
LA 30-Hz flicker	Peak	72.4 (19.8)	71.4 (6.6)	63.6 (25)	0.267
LA 3 (standard flash)	a-wave	24.0 (16.3)	22.4 (5.4)	20.5 (23.8)	0.010*
	b-wave	102.5 (1.7)	97.3 (0.7)	87.4 (1.4)	0.036*

DA refers to stimuli delivered in the dark following 20 min of dark adaptation; LA refers to stimuli delivered in the presence of the standard white photopic background following 10 min of light adaptation. *P* values in the final column are from a mixed linear model including all participants, using allelic dosage as a predictor and ERG parameters as outcomes, and adjusting for age, sex, and relatedness between individuals. *P < 0.05.

In Fig. 1*I*, averaged light-adapted flash ERGs from two of the three groups (no risk alleles versus two risk alleles) are compared. Averages from right and left eyes (continuous and dashed traces, respectively) are very similar within each group, consistent with the high overall reproducibility of the measurements. Responses from the two groups do not just differ in amplitude but waveform shape differs, with an inflection or gradient change seen in the red traces (group 2) at ∼25 ms postflash (blue arrow) that is not present in the black traces (group 0). This region of the waveform arises from postreceptoral activity in cone-driven ON and OFF pathways in the retina (16).

### ERG Responses to Experimental Protocols Isolating Dark-Adapted Rod and Cone System Responses.

In standard testing, although dark-adapted responses are largely rod-driven, responses to the standard and bright flashes (DA 3 and DA 10, respectively) contain significant contributions from stimulation of cone photoreceptors. The rod- and cone-mediated contributions to the ERG a-wave response to flashes delivered in the dark can be parsed by delivering identical flashes in the presence of a dim blue background (calculated to saturate the rods but minimally desensitize the cones) (17, 18). This yields dark-adapted cone-mediated responses; subtraction of these from responses to the same flashes in the dark yields an estimated dark-adapted rod photoreceptor response.

**Fig. 2.**
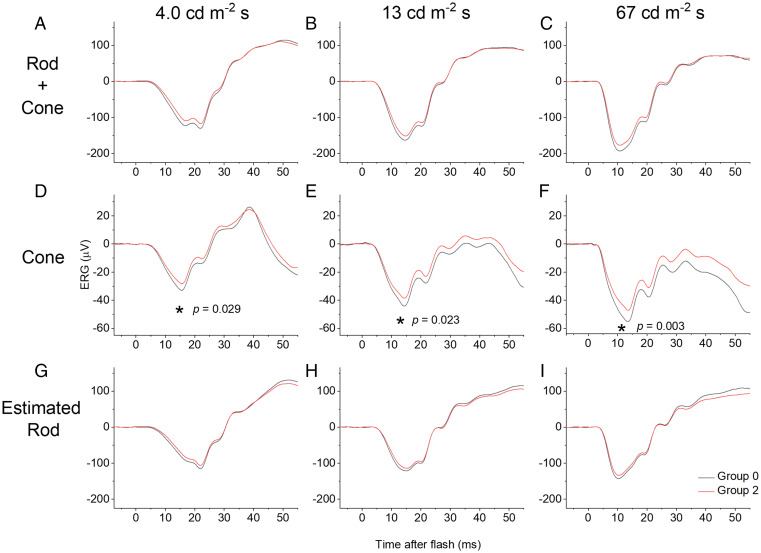
Averaged ERG responses to white flashes delivered in the dark (*A–C*) and on a rod-saturating blue background (*D–F*), and estimated dark-adapted rod-isolated responses (*G–I*). Black traces are from participants with no risk alleles (group 0) at the locus of interest; red traces are from participants homozygous for the risk allele (group 2). (*A–C*) Responses to white xenon flashes (photopic intensities shown) delivered in the dark. (*D–F*) Responses to identical flashes delivered on a blue background (30 scotopic and 1.0 photopic cd⋅m^−2^), chosen to saturate the rods but minimally affect the cones, and thus yield an estimate of the dark-adapted cone response. (*G–I*) Estimated isolated rod-driven responses obtained by numerical subtraction of traces in *D–F* from those in *G–I*. (Note that these traces might still contain a minor cone component if cones are slightly desensitized by the blue background.) Using the mixed linear model, a significant effect was observed across groups only for cone-driven a-wave amplitudes (denoted by asterisks in *D–F*).

Fig. 2 shows the results of this investigation: Averaged responses are shown for groups 0 and 2 for three different flash strengths delivered in the dark (Fig. 2 *A*–*C*; these are combined rod and cone system responses) and delivered in the presence of the rod-saturating blue background (Fig. 2 *D*–*F*; these are cone system responses). Fig. 2 *G*–*I* shows the result of subtracting the latter responses from the former, revealing an estimate of the isolated dark-adapted rod system response. Fig. 2 *D*–*F* (cone responses) shows a clear difference between the two groups, while the rod-isolated responses (Fig. 2 *G*–*I*) are similar. Table 2 shows the probabilities of association from the mixed linear model for estimated rod- and cone-isolated a-wave amplitudes: Of the four flash strengths delivered, three of the cone-driven a-waves show significant association (*P* < 0.01), while none of the rod-driven a-waves show a significant association. In multivariate analysis (*SI Appendix*, Table S2), the association with the cone-driven a-wave amplitude elicited by the brightest flash remained significant (*P* = 0.015).

**Table 2.** Mean (SD) a-wave amplitudes for rod and cone responses grouped by risk alleles

Background (rod or cone system)	Flash strength, photopic cd⋅m^−2^⋅s	Mean (SD) amplitudes, µV			*P* value (mixed model)
		No risk alleles (group 0), *n* = 37	One risk allele (group 1), *n* = 101	Two risk alleles (group 2), *n* = 48	
Rod-saturating blue background (CONE-DRIVEN RESPONSES)	0.67	17.2 (5.2)	16.0 (4.3)	14.9 (4.3)	0.152
	4.0	33.8 (8.9)	31.7 (7.3)	28.9 (7.5)	0.029*
	13	45.1 (10.1)	42.2 (8.6)	39 (8.4)	0.023*
	67	56.0 (13.0)	51.3 (11.3)	47.5 (9.8)	0.003*
Estimated ROD RESPONSES	0.67	77.6 (29.2)	74.9 (21.5)	68.6 (25.5)	0.574
	4.0	121.1 (31.9)	118.4 (26.9)	111.0 (28.4)	0.378
	13	125.7 (33.4)	123.8 (26.6)	115.7 (30.2)	0.355
	67	147.5 (36)	145.7 (29.1)	136.6 (30.8)	0.957

*P* values in the final column are from the mixed linear model. Responses on the blue background are from the cone system alone; here, all comparisons are significant except the dimmest flash. Subtraction of these responses from those to identical flashes delivered in the dark yields an estimate of the rod response; none of the comparisons were significant. As the blue background is still likely to mildly desensitize the cones, the subtraction might not be complete, and any residual trend in the rod response amplitudes might still be attributable to residual cone system components. The b-wave comparisons were not significant. Averaged responses are shown in Fig. 2 (except the dimmest flash). Comparisons for responses in the dark (Fig. 2 *A*–*C*) were not significant. *P < 0.05.

### Recordings from Patients with Loss of Bipolar Cells to Elucidate Components of Cone-Driven a-Waves and b-Waves.

Our findings indicate an association between cone-driven, but not rod-driven, electrophysiological responses and allelic identity at this locus. The differences are observable in the ERG a-wave, which is usually taken to reflect photoreceptor hyperpolarization secondary to shutoff of the cyclic nucleotide–gated (CNG) current in the photoreceptor outer segment. However, experiments in macaques (19, 20) have shown that the cone-driven a-wave also contains a substantial contribution from postreceptoral neurons, namely OFF-bipolar cells (which, like photoreceptors, hyperpolarize in response to light).

**Fig. 3.**
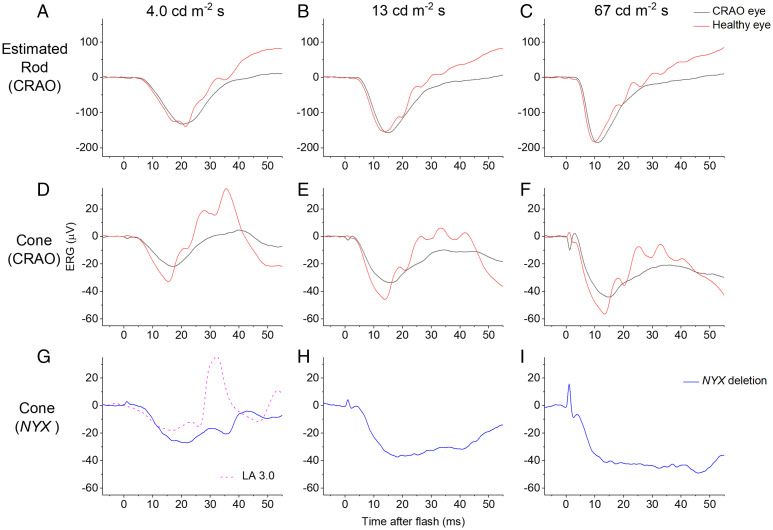
Rod and cone system responses from a patient with unilateral CRAO (*A*–*F*), and cone-driven responses from a male patient with *NYX* deletion (*G–I*). Stimuli are as in Fig. 2, with the same method of rod and cone isolation. (*A–C*) Rod system–isolated responses from a patient with CRAO. In the CRAO eye, inner retinal layers have degenerated but photoreceptors are intact: The a-waves are similar between eyes, reflecting intact rods, but b-waves differ, due to loss of inner retinal neurons. (*D–F*) Cone system responses from the same patient. As well as expected differences in b-waves due to loss of inner retinal layers, a-wave amplitudes also differ markedly, consistent with a postreceptoral origin of a substantial fraction of the cone-driven a-wave. (*G–I*) Cone-driven responses to the same flash strengths from a patient with selective loss of ON-bipolar cell signals. The b-wave is severely attenuated in the responses shown (particularly brighter flashes), indicating that the normal b-wave elicited by these stimuli in healthy individuals is from the ON pathway. The dashed trace shows standard LA 3 response in this patient (same stimulus as in Fig. 1 *G–I*): A b-wave is present, indicating that for this stimulus, OFF-bipolar cells do contribute to the b-wave.

We investigated this possibility by analyzing responses to the same rod- and cone-isolating stimuli (as those shown in Fig. 2) from a patient in whom postreceptoral neurons had been largely lost in one eye secondary to a prior CRAO. Following CRAO, inner retinal neurons are lost but the photoreceptors (supplied by the choroidal circulation) are preserved (confirmed in this patient with optical coherence tomography imaging; *SI Appendix*, Fig. S3). Fig. 3 *A*–*F* shows responses from this patient, comparing the CRAO eye with the healthy fellow eye. The b-wave is diminished in the CRAO eye to all stimuli as expected. The rod-isolated a-wave amplitude is similar between the two eyes but the cone-driven a-wave is diminished in the CRAO eye, supporting the notion that a substantial part of this response in humans (as in macaques) is indeed postreceptoral in origin. Thus, the differences observed in the cone-driven a-wave in Figs. 1 and 2 might well be attributable to alterations in postreceptoral activity.

Our previously described genetic analyses suggested that the international standard light-adapted cone-driven b-wave amplitude associated significantly with allelic identity at this locus (Fig. 1), but cone-driven b-wave amplitudes elicited by flashes delivered on the dim blue background showed no such association. To understand the origin of the b-wave in these different stimulus paradigms, we analyzed cone-driven responses from a patient with molecularly confirmed *NYX*-related cCSNB. This condition entails selective loss of ON-bipolar cell signals and intact OFF-bipolar cell signals (21). Cone-driven ERGs from this patient elicited by the same flashes are shown in Fig. 3 *G*–*I*. Here, there is marked loss of the b-wave in the responses to flashes delivered on the dim blue background, indicating that in healthy individuals this component arises from ON-bipolar cells. However, this patient did show a clear b-wave in response to the standard light-adapted flash (dashed violet trace in Fig. 3*G*), consistent with a contribution from OFF-bipolar cells (as this patient has no ON-bipolar cell responses). *SI Appendix*, Fig. S4 depicts schematically the impairments in signaling in the two types of patient. These findings would suggest that the particular associations we have found with the myopia locus are all consistent with differences in the cone-driven OFF-bipolar cell response.

We also analyzed responses from a second patient with cCSNB from another genetic cause (biallelic pathogenic variants in the *TRPM1* gene) (22–25). This patient showed the same shape of responses to the various stimuli as seen in the patient with *NYX*-associated cCSNB (illustrated in *SI Appendix*, Fig. S5), confirming that such waveforms are a general feature of selective loss of the ON-bipolar contribution, and that a clear b-wave is present in response to the standard light-adapted flash. *SI Appendix*, Fig. S6 shows averaged responses to the standard light-adapted flash from the healthy participants of groups 0 and 2 (replotting the data from Fig. 1*I*), and also the responses to the same stimulus from the patients with loss of ON-bipolar signals. The start of the b-wave in the patients occurs at a similar poststimulus time to the time window over which the b-waves of groups 0 and 2 differ, which would be consistent with the notion that OFF-pathway signals are contributing to this difference.

Further testing of this hypothesis is illustrated in *SI Appendix*, Fig. S7. Here, averaged cone-driven ERGs from groups 0 and 2 (elicited by flashes delivered on the blue background; Fig. 2 *D*–*F*) are shifted on the *y* axis by the amplitude of the a-wave, so that comparison can be made specifically with respect to the b-waves. The b-waves coincide for the brighter flashes, and nearly coincide for the dimmer flash. *SI Appendix*, Fig. S7 *D*–*I* replots the traces from the patients with loss of ON-bipolar signals (as in Fig. 3 *G*–*I* and *SI Appendix*, Fig. S5 *D*–*F*) for direct comparison. The response to the dimmer flash strength in these patients does show some upward deflection after the a-wave trough coinciding with the timing of the b-waves in *SI Appendix*, Fig. S7*A*, consistent with the notion that any differences between the two waveforms in *SI Appendix*, Fig. S7 *A*–*C* arise from cone-driven OFF-pathway activity.

### Transcriptome Studies of *GJD2* Cellular Expression in Human Retina.

**Fig. 4.**
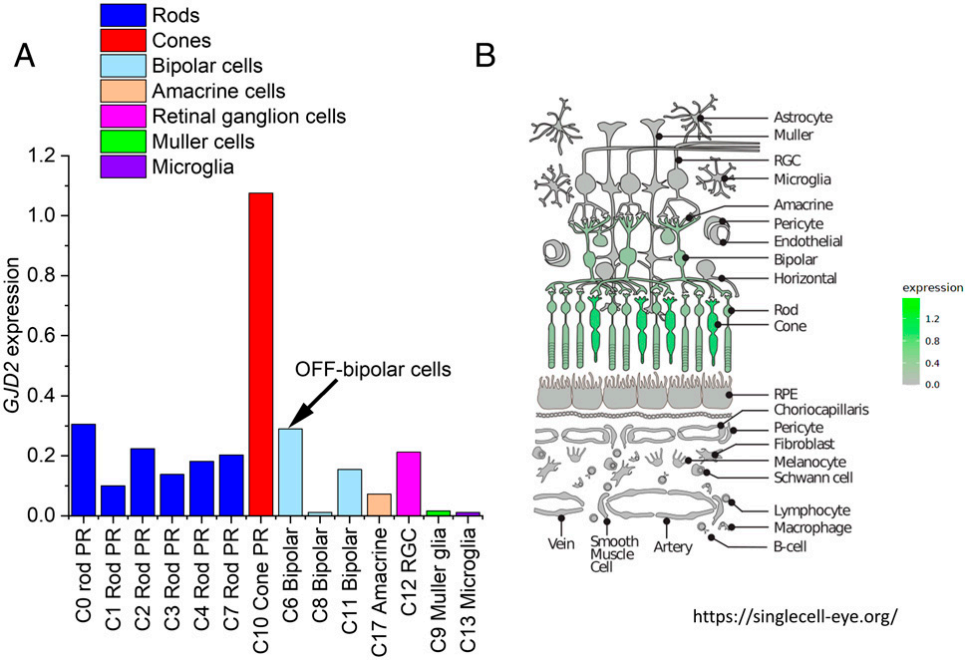
Retinal expression of *GJD2* from two human single-cell transcriptome studies. (*A*) Data plotted from a supplementary table of a published study from three human donors (26). Eighteen transcriptionally distinct clusters (C0 to C17) were reported. Data plotted here are for *GJD2* expression levels in 14 clusters (data for the following clusters are not shown: C15 [horizontal cells] and C16 [astrocytes] had no *GJD2* expression; C5 [others] and C14 [others] are also omitted, as the cell type was not identifiable). The C6 (bipolar) cluster was identified as OFF-bipolar cells due to high expression of *GRIK1*. (*B*) Retinal schematic highlighting cells expressing *GJD2* from another single-cell transcriptome dataset (the Spectacle online resource, accessible at https://singlecell-eye.org/). Expression levels are highest in cones, with significant expression also in rods and bipolar cells. PR, photoreceptor; RGC, retinal ganglion cell; RPE, retinal pigment epithelium.

Fig. 4*A* plots *GJD2* expression levels for the transcriptionally distinct clusters found by Lukowski et al. (26); the clusters have been reordered to show similar cell types near each other. The highest expression by far is in cones. There is some expression in rods. There is significant expression in bipolar cells and, of the bipolar cell clusters, the greatest expression is in cluster C6, which they identified as OFF-bipolar cells (as this cluster showed the highest expression of the OFF-bipolar cell marker *GRIK1*). The C8 cluster (which is the bipolar cell type showing the lowest *GJD2* expression) was identified as rod-driven ON-bipolar cells (expressing the marker *PRKCA*), while the C11 cluster was identified as a population of cone-driven ON-bipolar cells. Fig. 4*B* depicts data derived from another single-cell transcriptome dataset (including human retina, retinal pigment epithelium, and choroid) (27). The schematic shows the main retinal cell types, and the color intensity correlates with *GJD2* expression. Again, highest expression is seen in cone photoreceptors, with significant expression also seen in rods and bipolar cells.

## Discussion

We analyzed retinal electrophysiological responses to both standard and customized experimental stimulus protocols in 186 healthy genotyped participants to investigate associations between ERG parameters and a common myopia-associated locus. As this locus is close to the *GJD2* gene, which encodes retinal gap junctions (connexin-36), we hypothesized that allelic identity might associate with one or more electrophysiological parameters. We have previously found standard ERG parameters to show high heritability (15) and, in the present study, we adjusted for interindividual relatedness (as well as age and sex). When analyzing international standard parameters, significant associations were found with light-adapted flash ERG a-wave and b-wave amplitudes (and with light-adapted a-wave and flicker ERG amplitudes in multivariate analysis) but not with dark-adapted ERGs, suggesting that cone-driven signals may be associated with allelic identity.

The shape of the waveform showed a difference between those with no risk alleles and those homozygous for the risk allele, with an inflection seen in the response at ∼25 ms in the latter group. This region of the waveform arises from postreceptoral activity in cone-driven ON and OFF pathways in the retina. Differences could arise from changes in the relative strength of signals in these pathways, or their synchronization, or indeed from faster oscillatory potentials arising from amacrine cells.

Further examination of responses to experimental ERG protocols designed to separate dark-adapted rod and cone responses confirmed an association with cone-driven responses: Cone-driven a-wave amplitudes for three of four flash strengths associated significantly with identity at the locus of interest, suggesting that any apparent differences in dark-adapted ERGs (Fig. 1 *A*–*E* does show a possible dose–response, though not significant) are likely to originate in the cone-driven, rather than the rod-driven, contribution to the dark-adapted ERG.

Immunohistochemistry studies in human retina have demonstrated connexin-36 in outer and inner plexiform layers (10): In the outer plexiform layer, which contains synapses between photoreceptors, bipolar cells, and horizontal cells, connexin-36 has been localized to cone photoreceptor axons, cone terminals, and cone bipolar cell dendrites (10). This pattern of localization would be consistent with effects on responses of second-order neurons (ON- and OFF-bipolar cells) but would not be expected to alter the CNG current in the photoreceptor outer segment, and so it may be puzzling initially to see an effect in the ERG a-wave.

Although the a-wave is conventionally attributed to photoreceptor signals (photoreceptor outer-segment phototransduction resulting in the closure of CNG channels, causing hyperpolarization of the photoreceptor), the cone-driven a-wave has been shown to contain signals from inner retinal cells, namely OFF-bipolar cells, in primate models (19, 20). We showed that this is also likely to be the case in the human ERG: In recordings from a patient with loss of inner retina in one eye secondary to a previous CRAO, isolated rod-driven a-waves were near-identical between the two eyes, while cone-driven a-waves were of lower amplitude in the CRAO eye, indicating that some of the a-wave comes from postreceptoral (likely OFF-bipolar) cells; thus, allelic identity at the locus of interest might be specifically affecting signals generated by these cells. The association of lower amplitudes with the presence of the myopia risk allele might indicate that this allele associates with less hyperpolarization of OFF-bipolar cells for a given stimulus.

As mentioned, the light-adapted standard flash b-wave amplitude associated with allelic identity at the locus of interest. However, the dark-adapted cone-driven b-wave amplitudes (elicited by flashes delivered on a dim rod-saturating blue background) did not show a significant association; they were near-identical across the different groups. Responses to the same stimuli from patients with selective loss of ON-bipolar cell signals revealed that, while the standard light-adapted flash b-wave contains significant OFF-bipolar cell signals, these are largely absent from the responses to the stimuli delivered on the blue background, particularly for brighter flashes. Although a limitation of this analysis is that it was performed in only two patients (such diseases are rare), the findings were reproducible despite the patients having different genetic causes for their loss of ON-bipolar signals. The findings are again consistent with any difference between groups in our study being attributable to cone-driven OFF-pathway signals. Previous studies analyzing responses to the stimuli on white backgrounds have also shown differences in relative ON and OFF contributions to the b-wave depending on flash strength (16), and have shown a clear b-wave in response to the standard light-adapted flash in patients with loss of ON signals (13, 16, 21, 22).

Other factors that can affect ERG amplitudes include age (15, 28), axial length (29), and electrode position (30). However, these would not be expected to selectively affect cone-driven over rod-driven responses, or to selectively affect a-waves rather than b-waves. Mean ages did not differ between groups, and electrode position was consistent and continually checked. Mean (SD) axial lengths did not differ significantly between groups, suggesting the alterations in retinal electrophysiology are not explained by axial length. On the contrary, we hypothesize that alterations in electrophysiology contribute, together with numerous other factors, to altering the drive for eye growth.

Our findings specifically suggest an effect on the strength of signals in the cone-driven OFF pathway, presumably due to a change in levels of expression of the nearby *GJD2* gene, encoding connexin-36. Whether the electrophysiological changes would reflect increased or decreased *GJD2* expression is not known. Immunohistochemical studies have already demonstrated the presence of the protein in the outer plexiform layer, and our exploration of single-cell transcriptome data from two different datasets showed consistent results: *GJD2* is most highly expressed in cones; there is also expression in rods and in bipolar cells and, of the latter, the strongest expression appears to be in OFF-bipolar cells and the least in rod-driven ON-bipolar cells.

Higher environmental light levels (usually found outdoors rather than indoors) are protective for myopia (31, 32), and these intensities tend to be in the photopic, rather than scotopic, range. At these levels, variations are expected in cone responses, while rod photoreceptors are usually saturated. The results of the present study similarly appear to highlight changes in cone-mediated, rather than rod-mediated, signaling. This does not exclude scotopic signaling having a role in myopia; it is possible that such associations might be found with other loci or might even emerge with respect to this locus in a much larger study, powered to show very small effects. The findings for our locus of interest were internally consistent: Light-adapted cone-driven OFF-pathway signals are common generators for all of the parameters that emerged as significantly associated with allelic identity.

Data for *GJD2* expression from other tissues (including skeletal muscle, pituitary, pancreas, and heart tissues) indicate that expression levels do differ with genotype at the rs524952 locus (https://gtexportal.org/home/snp/rs524952, accessed 6 March 2022). The relationship between this locus and retinal expression levels of *GJD2* might be revealed by future detailed studies focusing on retinal expression.

The age of our cohort is well beyond the time period at which myopia commonly develops, and it is not certain that our findings would be representative of electrophysiological changes at younger ages. Future studies in larger cohorts, and in younger age groups, might help answer these questions, and might be powered to detect significant associations in other ERG parameters (for example, peak times of ERGs, amplitudes of oscillatory potentials, and rod-driven signal parameters) with allelic variants at this locus or other loci associated with myopia. Additional experimental protocols might shed further light on potential retinal signaling pathways driving eye growth and refractive error. For example, dopaminergic pathways appear to be important in both retinal light adaptation and myopia (33, 34). Electrophysiological studies specifically tracking kinetics of retinal light and dark adaptation and investigating associations with myopia risk loci could be particularly informative.

Several studies have investigated aspects of retinal ON- and OFF-pathway signaling in relation to myopia (35–39), and some studies have shown different effects of visual stimuli (favoring ON or OFF stimulation, respectively) on choroidal thickness (38, 39), which itself is associated with myopia. Over 30 y ago, blockade of ON-channel activity in kitten eyes was shown to lead to hyperopia (35), indicating an inhibition of axial elongation. Other studies have shown ostensibly opposing findings: Congenital loss of the ON pathway leads to greater susceptibility to myopia (36). Pardue et al. found that mice with *nyx* mutations were initially more hyperopic than wild-type mice, but were more susceptible to form deprivation myopia (36). In humans with congenital ON-pathway loss (complete CSNB), high myopia is usually seen; however, *CACNA1F*-associated disease (sometimes termed “incomplete” CSNB), which entails attenuation of both ON- and OFF-pathway signals, is also associated with myopia (25). Mechanisms driving myopia in the general population are likely to relate to more subtle aspects of the balance between ON- and OFF-pathway signaling, possibly at particular periods in development, rather than profound global attenuation of one pathway or the other.

Studies have also shown intriguing differences in ERGs that appear to be associated with myopia (40–44), with differences demonstrated in the multifocal ERG (40–43). In the present study, the aim was not to explore associations with myopia itself but with the particular polymorphism near *GJD2*, with the broader aim that this could help elucidate the functional consequence of the different alleles. This might in turn shed light on how this locus might confer myopia risk, though further work is needed to establish causality. It is likely that other genetic associations will act through other mechanisms.

In summary, taken together, our findings suggest associations between photopic cone-driven (but not necessarily scotopic rod-driven) retinal electrophysiological signals and one of the genetic polymorphisms most strongly linked to myopia. Axial length elongation is known to be driven by retinal signaling, and this finding supports the hypothesis that alterations in light-adapted cone system signaling, in particular those affecting balance between ON and OFF pathways, may contribute to myopia development.

## Materials and Methods

### Participant Recruitment.

Participants were recruited from the TwinsUK cohort by telephone invitation. Participants were all adults, and were only excluded from recordings if they were unable to undergo pharmacological pupil dilation (for example, in cases of known iridocorneal angle closure) or if there was a history of photosensitive epilepsy. Two hundred and ten participants underwent recordings (105 twin pairs) for a study initially aiming to investigate heritability of ERG parameters (15). Those without relevant genotypic information were excluded from the present study. In addition, three patients (unilateral CRAO and cCSNB) were recruited from specialist retinal clinics. The aims, procedures, and risks of the study were explained, and participants gave written informed consent.

### Ethical Approval.

The study had local ethics committee approval (National Health Service Health Research Authority Research Ethics Service, Research Ethics Committee London–Harrow, Reference 11/LO/2029) and conformed to the tenets of the Declaration of Helsinki.

### ERG Recordings.

Pupils were pharmacologically dilated (using topical 1.0% tropicamide and, in most cases, 2.5% phenylephrine). The skin was cleaned using alcohol wipes prior to placement of skin electrodes (24-mm disposable ground electrodes; Unimed Electrode Supplies Limited) on the temples (reference electrodes) and forehead (ground electrode). Conductive fiber electrodes (DTL-PLUS electrode; Unimed Electrode Supplies Limited) were placed deep in the lower conjunctival fornix (position was checked regularly). Full-field stimuli and recording were performed using ColorDome with Espion software (Diagnosys), incorporating the standard International Society for Clinical Electrophysiology of Vision (ISCEV) protocol for full-field electroretinography (10). Stimuli were delivered via an integrating sphere, which is painted white internally, to allow uniform stimulation of the retina. Participants were dark-adapted for 20 min prior to delivery of scotopic stimuli. Standard scotopic stimuli were delivered, comprising white flashes of 0.01, 3.0, and 10.0 photopic cd⋅m^−2^⋅s (light-emitting diode [LED] stimuli). Participants were then exposed to xenon flashes of the following strengths: 0.67, 4.0, 13, and 67 photopic cd⋅m^−2^⋅s, delivered first in the dark, to elicit combined rod and cone system responses, and then in the presence of a blue rod-saturating LED background (30 scotopic cd⋅m^−2^ and 1 photopic cd⋅m^−2^), to elicit responses solely from the cone system. Participants were subsequently exposed to the standard ISCEV photopic white light-adapting background (30 photopic cd⋅m^−2^) and underwent standard ISCEV photopic recordings (in response to the 30-Hz flicker stimulus and the standard photopic flash of 3.0 cd⋅m^−2^⋅s) following at least 10 min of light adaptation. Responses were averaged from multiple stimulus presentations (interstimulus intervals ranged from 5 to 30 s for dark-adapted stimuli, and between 0.5 and 5 s for photopic stimuli).

### ERG Analysis.

Responses containing excessive noise or artifact were rejected as previously described (15, 17). Responses were averaged over a 20-ms time window prior to flash delivery to derive the baseline. Response parameters were averaged from both eyes. The experimental protocols permitted separation of rod- and cone-driven components of the dark-adapted flash responses. The responses to flashes delivered on the dim blue rod-saturating background reflected the dark-adapted cone-driven response. Mathematical subtraction of these responses (cone-driven) from those to identical flashes delivered in the dark (rod- and cone-driven) yielded the isolated dark-adapted rod-driven response (i.e., with the cone-driven components removed) (18).

### Axial Length Measurements and Autorefraction.

Ocular axial lengths were measured using noncontact optical biometry (IOLMaster 500 v.7.5, Carl Zeiss Meditech, or NIDEK AL-Scan Optical Biometer v.1.06.01, Nidek) performed by the same operator (right eyes measured first). Axial lengths were averaged from both eyes. The majority of participants had also undergone measurement of refractive error by autorefraction (Humphrey-670, Humphrey Instruments, or ARM-10, Takagi Seiko). Spherical equivalent refractions were averaged from both eyes.

### Genotyping.

Genotyping of the TwinsUK cohort was done using Illumina HumanHap610Q chips. Intensity data were called using the proprietary GenomeStudio v.2.0 calling algorithms. Imputation was performed using the Minimac3 software package (45) using haplotypes from Haplotype Reference Consortium r1.12016 as a reference (46).

### Statistical Testing.

Association testing was performed using all of the genotyped subjects and a mixed linear model, which adjusts for intrafamilial relatedness, as implemented in the software GEMMA (47), where the electrophysiological parameters served as outcomes and allelic dosage at the rs524952 locus as a predictor. Adjustment was made for age, sex, and interindividual relatedness (these were used as covariates). We defined statistical significance as any association with probabilities *P* < 0.05. Although a number of ERG parameters were examined, several were correlated; the parameters fell essentially into two groups, namely dark-adapted and light-adapted. Hence, no formal correction for multiple testing was performed in the main analysis.

For further analysis, we built a multivariate association model with the aim of testing for associations of the genetic variant of interest jointly with the standard ERG amplitude parameters. For this purpose, we used MTAG, a software tool for multitrait analysis of genetic results (48). This analysis sets various parameters, such as genetic correlation between traits and matrices of variance–covariance of genetic effects and estimated errors across the entire genome. For this purpose, summary statistics of genome-wide association results for the ERG parameters were used, although the multivariate results were for only the predefined single-nucleotide polymorphism of interest (rs524952).

When investigating the experimental ERG parameters, a similar approach was used. However, likely owing to the small sample size relative to the correlation between some ERG parameters, a large multivariate model including parameters characterizing both cone and rod responses failed to converge. We subsequently split the ERG parameters into two groups corresponding to cone-driven and rod-driven responses, respectively, and ran two multivariate analyses. Both models converged, and significant associations are highlighted in *Results*.

### Analysis of Prior Single-Cell Transcriptome Studies.

We investigated prior single-cell transcriptome studies from human retina to identify the cell types in which *GJD2* was significantly, or most, expressed. Lukowski et al. undertook single-cell RNA sequencing from over 20,000 cells from 3 human donors, finding 18 cell populations that were transcriptionally distinct (26). In their supplementary data files, they give expression levels of over 20,000 genes in these clusters (C0 to C17); we interrogated this dataset for *GJD2*. The second transcriptome dataset we accessed was the “Spectacle” resource available online (https://singlecell-eye.org/, accessed 20 September 2021), developed at the University of Iowa Institute for Vision Research (27). We used the “all_retina_RPE_chor” dataset to generate a heatmap for *GJD2* expression.

## Supplementary Material

Supplementary File

## Data Availability

Relevant data are given within the manuscript and/or *SI Appendix*. Some study data are available (applications can be made for access to data relating to the TwinsUK resource as detailed here: https://twinsuk.ac.uk/resources-for-researchers/access-our-data/).
