# Supplementary material for: Electrical responses from human retinal cone pathways associate with a common genetic polymorphism implicated in myopia
Source: Proc Natl Acad Sci U S A. 2022 May 20;119(21):e2119675119. doi: 10.1073/pnas.2119675119 (PMC9173800; doi:10.1073/pnas.2119675119)
Supplement: Supplementary File [file pnas.2119675119.sapp.pdf]

PNAS

Supplementary Information for
Electrical responses from human retinal cone pathways associate with a common genetic polymorphism implicated in myopia

Xiaofan Jiang MSc,[a-c] Zihe Xu MSc,[b,c] Talha Soorma MB BS,[b] Ambreen Tariq MSc,[b,c] Taha Bhatti MSc,[b,c] Alexander J Baneke MB BS,[b] Nikolas Pontikos PhD,[a] Shaun M. Leo MSc,[a,d] Andrew R Webster MD(Res), FRCOphth,[a,d] Katie M Williams PhD, FRCOphth,[a-d] Christopher J Hammond MD(Res), FRCOphth,[b,c] Pirro G Hysi PhD,[b,c]* Omar A Mahroo PhD, FRCOphth[a-e]*

[a]Institute of Ophthalmology, University College London, Bath Street, London, EC1V 9EL, United Kingdom

[b]Department of Ophthalmology, King's College London, St Thomas' Hospital Campus, London, SE1 7EH, United Kingdom

[c]Department of Twin Research and Genetic Epidemiology, King's College London, St Thomas' Hospital Campus, London, SE1 7EH, United Kingdom

[d]Medical Retina Service and Inherited Eye Disease Service, Moorfields Eye Hospital, London, EC1V 2PD, United Kingdom

[e]Physiology, Development and Neuroscience, University of Cambridge, Cambridge, CB2 3EG, United Kingdom

*Corresponding authors: Omar Mahroo (o.mahroo@ucl.ac.uk), Pirro Hysi (pirro.hysi@kcl.ac.uk)

This PDF file includes:

Figures S1 to S7

Tables S1 to S2

Fig. S1.

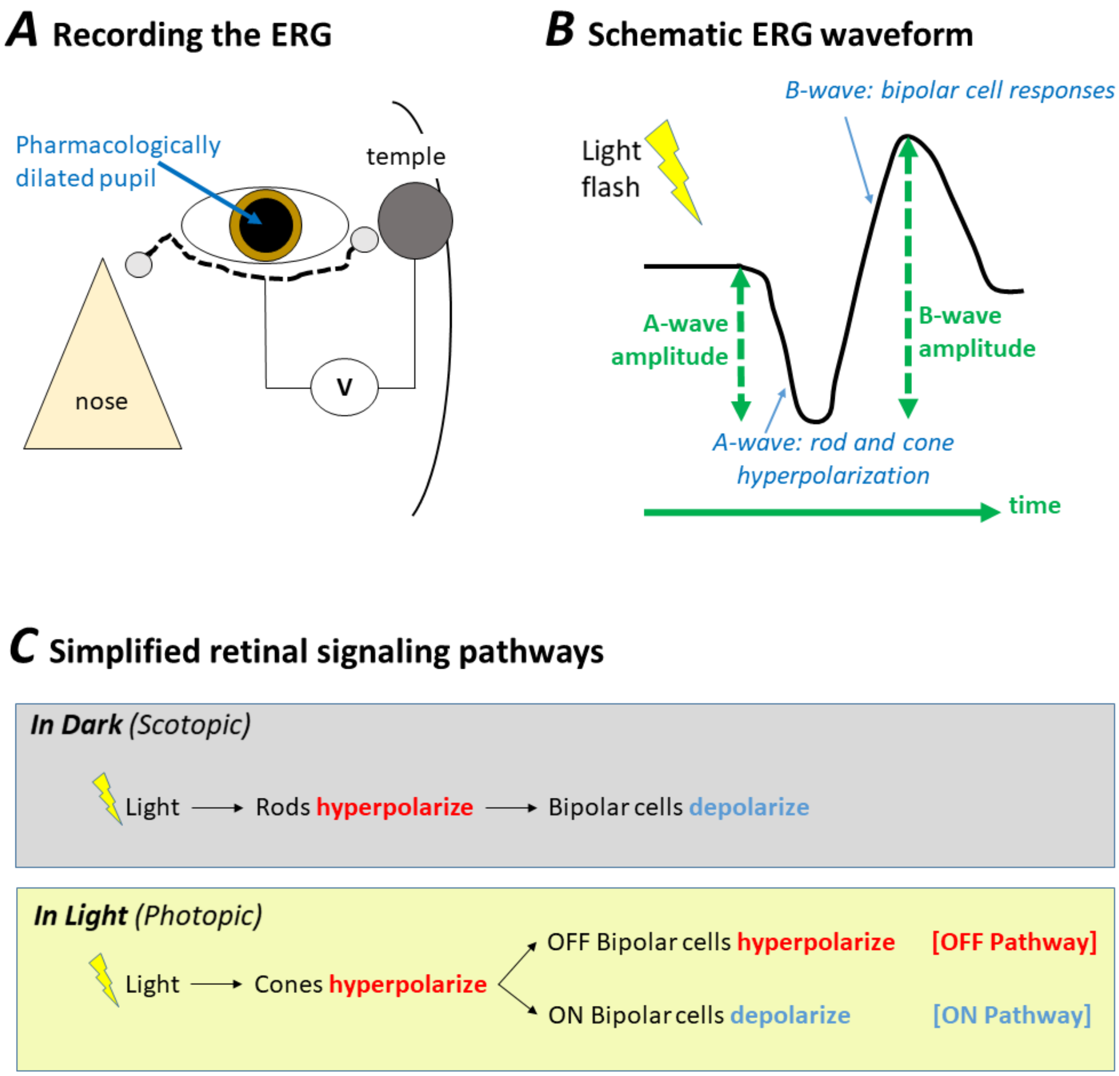

Figure S1. Simplified illustration of electroretinogram (ERG) recording and major origin of signals in dark and light. *A*, A conductive fibre electrode (dashed black line) is placed in the lower conjunctival fornix, and the potential difference is measured between this and an indifferent skin electrode (large grey circle) placed on the temple. A ground electrode (not shown) is placed on the forehead. *B*, Schematic ERG response to a flash. The initial negative component (termed the a-wave) arises largely from hyperpolarization from rod and cone photoreceptors. The subsequent positive component (b-wave) arises largely from bipolar cells. *C*, the major signaling neurons (first two neurons in the visual pathway) in light and dark, giving rise to the components in *B*. Rods synapse with depolarizing (ON) bipolar cells only, whilst cones synapse with both depolarizing (ON) and hyperpolarizing (OFF) bipolar cells. The cone system makes a contribution in the dark also. Supplementary Fig. 2 depicts cellular origins of standard dark-adapted and light-adapted ERGs in more detail.

Fig. S2.

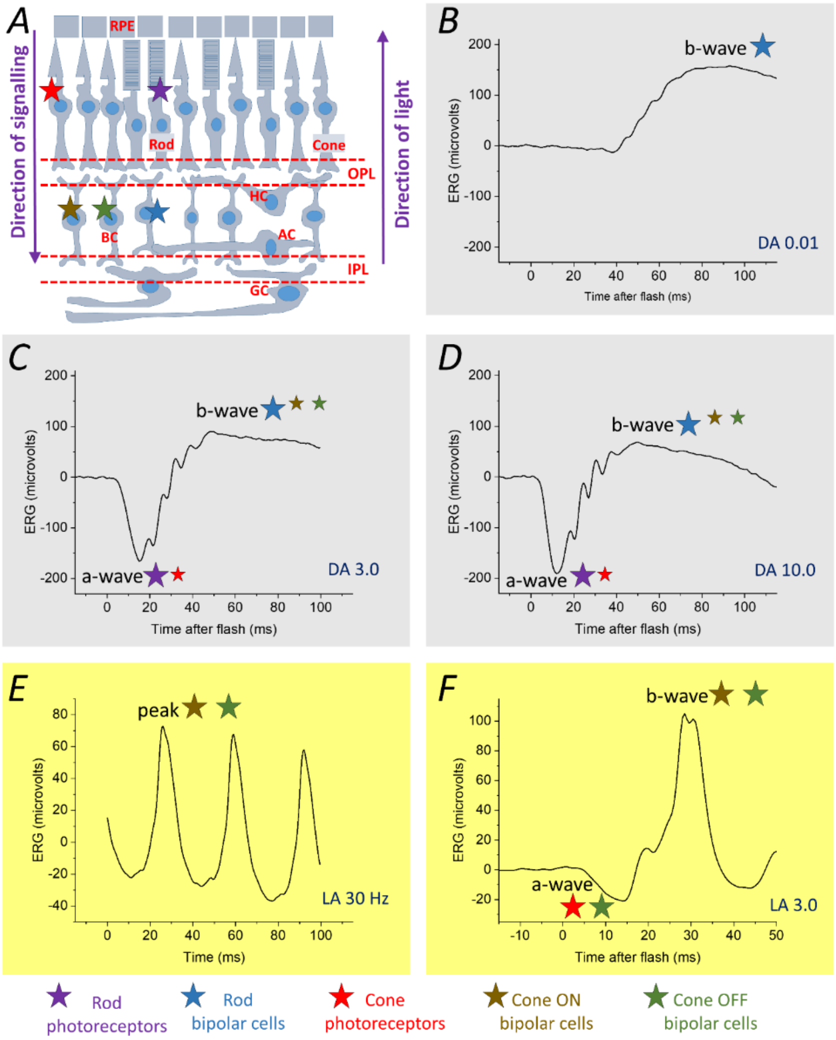

Figure S2. Retinal neurons and electroretinogram (ERG) responses to standard stimuli. *A*, Retinal schematic: BC, bipolar cell; HC horizontal cell; AC, amacrine cell; GC, ganglion cell; RPE, retinal pigment epithelium; OPL, outer plexiform layer; IPL, inner plexiform layer. Photoreceptors hyperpolarize in light: cones respond at higher illumination, synapsing with ON (depolarizing) and OFF (hyperpolarizing) bipolar cells; rods sense photons at dimmer levels, synapsing with rod-driven (ON) bipolar cells, whose signals feed into cone bipolar pathways via amacrine cells. Gap junctions mediate electrical coupling in plexiform layers. *B-F*, ERGs to standard stimuli. Stars indicate likely cellular origins. Grey panels show dark-adapted responses (mainly rod system) following 20 min dark adaptation. Yellow panels show light-adapted responses (cone system) in 30 cd m^{-2} white background following 10 min adaptation. DA 0.01, DA 3.0, DA 10.0 denote 0.01, 3.0 and 10.0 cd m^{-2} s white flashes (dark-adapted). LA 30 Hz and LA 3.0 denote white 30 Hz flicker and 3.0 cd m^{-2} s flashes (light-adapted; all units photopic). In flash responses, amplitudes of initial negative-going a-wave, and subsequent positive b-wave, are measured: a-wave amplitudes are baseline to trough; b-wave amplitudes are measured from a-wave trough to b-wave peak.

Fig. S3.

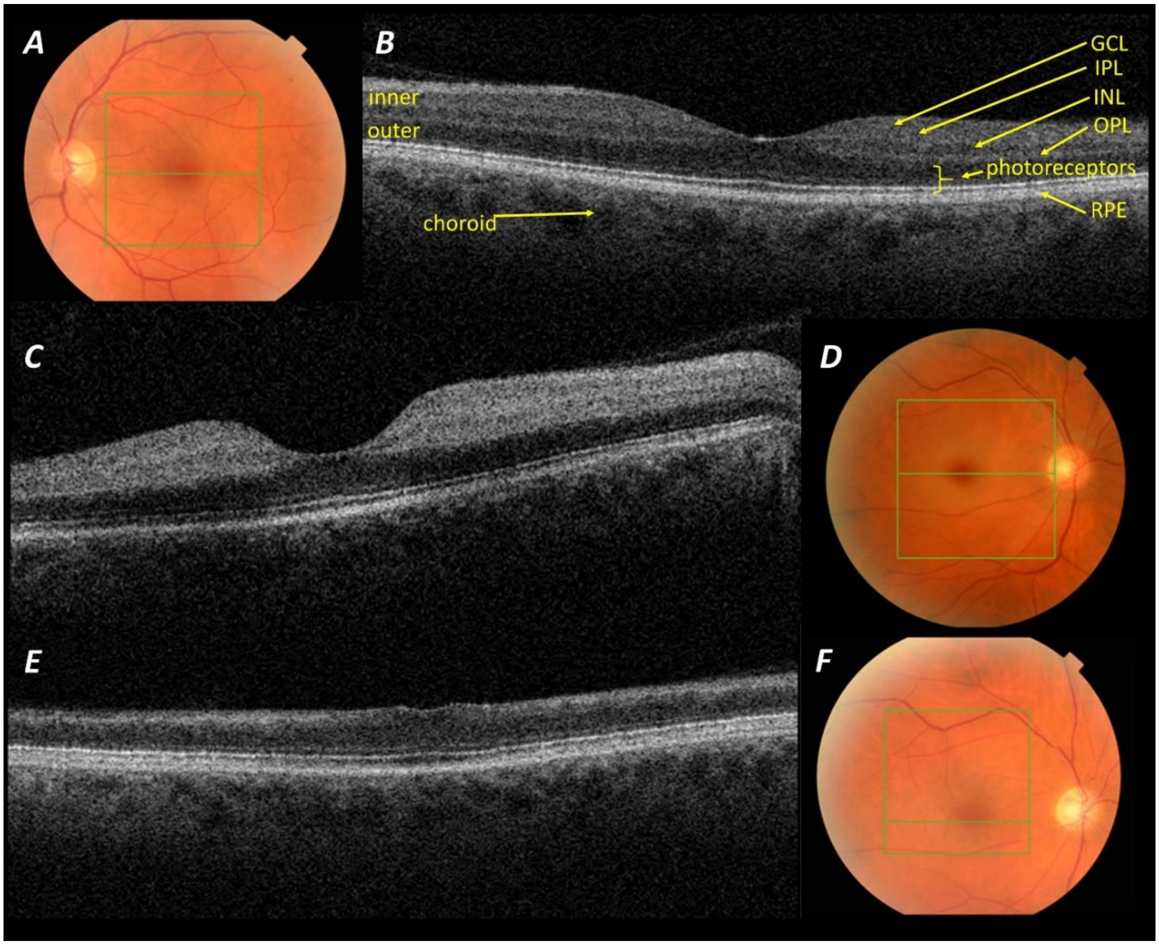

Figure S3. Cross-sectional retinal images from patient with unilateral central retinal artery occlusion. *A*, Colour retinal photograph of healthy left eye. *B*, Spectral domain optical coherence tomography (OCT) image of the same eye showing the retinal layers in cross section. The horizontal scan is through the foveal centre corresponding to the green line in *A*. Inner and outer retinal layers are labelled: specific layers labelled are GCL (ganglion cell layer); IPL (inner plexiform layer); INL (inner nuclear layer, containing nuclei of bipolar cells, horizontal cells and amacrine cells); OPL (outer plexiform layer); photoreceptors; RPE (retinal pigment epithelium). *C*, OCT image of right eye affected acutely by central retinal artery occlusion. The inner retinal layers are swollen and hyper-reflective. *D*, Colour retinal photograph showing perifoveal pallor and classic central “cherry red spot”. *E*, OCT image and photograph of the same eye after 4 months, showing atrophy of the inner retinal layers with preservation of the outer retina (photoreceptors and RPE). *F*, Colour retinal photograph at the same time point showing optic disc pallor. The ERGs in Fig. 3 in the main text were recorded from this patient 2.5 months after the acute event.

Fig. S4.

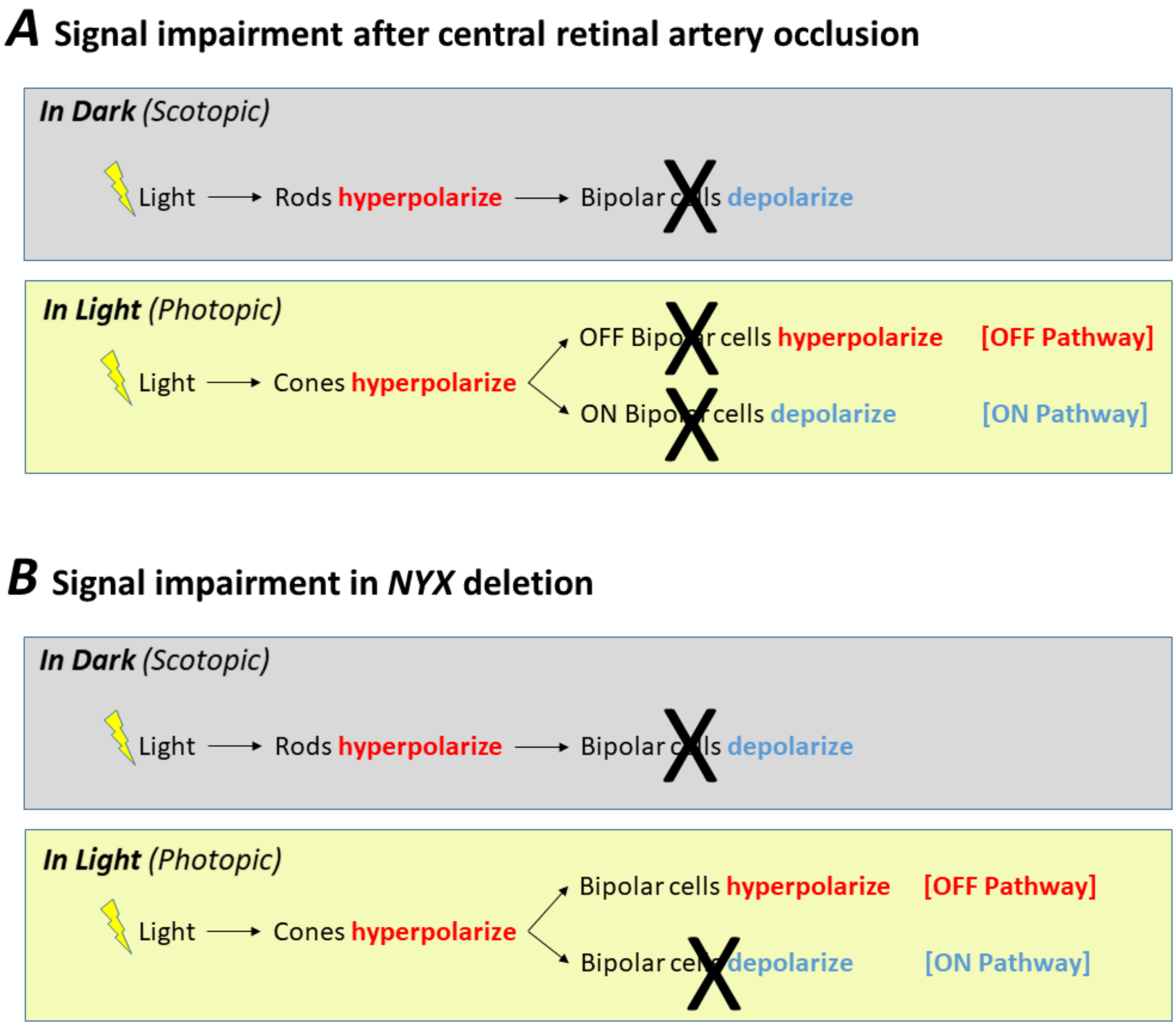

Figure S4. Signal impairments in patients. *A*, In the patient who had suffered a prior central retinal artery occlusion, the rod and cone photoreceptors are still viable (as they are supplied by the choroidal circulation), whilst the bipolar cells (and other inner retinal neurons) have been largely lost in the affected eye. Thus, the ERG elicited by light flashes will contain signals that primarily derive from photoreceptors. *B*, In the patient with a deletion in the *NYX* gene (the protein product is an important component of ON bipolar cell signalling), responses from depolarizing ON-bipolar cells have been selectively lost. The ERGs shown in Fig. 3 in the main text are interpreted in light of this. The same defect will occur in the patient with *TRPM1*-associated impairment.

Fig. S5.

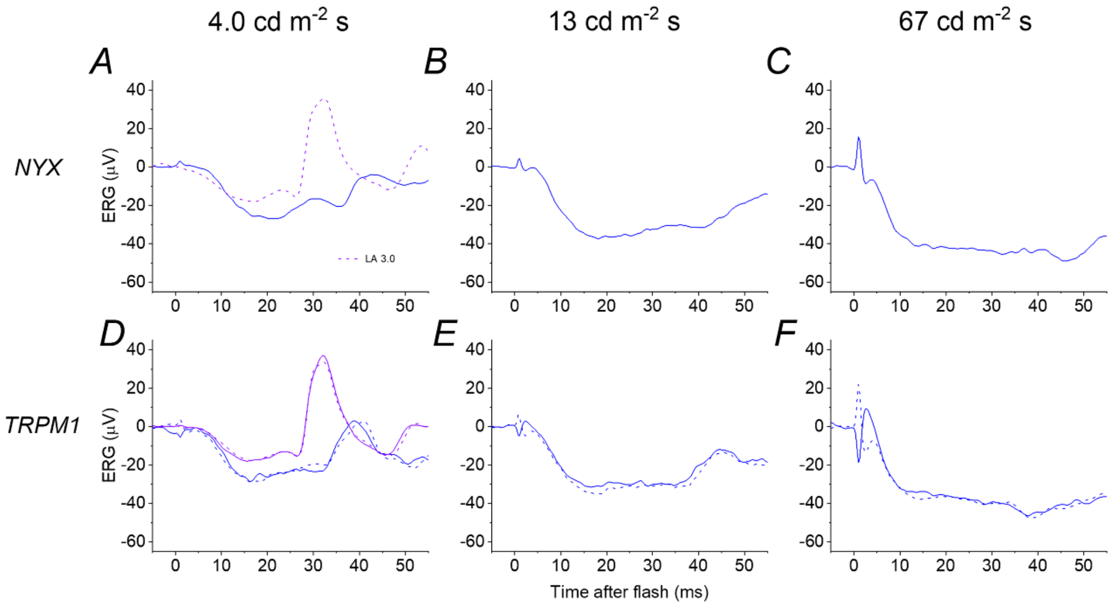

Figure S5. Comparison of cone-driven ERGs in two patients with loss of ON bipolar signals. *A-C*, These panels show the same ERGs depicted in Fig. 3 in the main text (lower panels) from a male patient with *NYX*-related X-linked complete congenital stationary night blindness (cCSNB). *D-F*, Responses to similar stimuli recorded from a female patient with autosomal recessive cCSNB associated with bi-allelic variants in the *TRPM1* gene. The LA3 flash stimuli and the other stimuli in D and E were elicited by LEDs; all other stimuli were elicited by xenon flashes. The dashed traces in the lower panels show responses from the patient's left eye; the solid traces are from the right eye. The waveforms in the upper and lower panels are similar, illustrating features of loss of ON bipolar cell signals although the genetic causes are different.

Fig. S6.

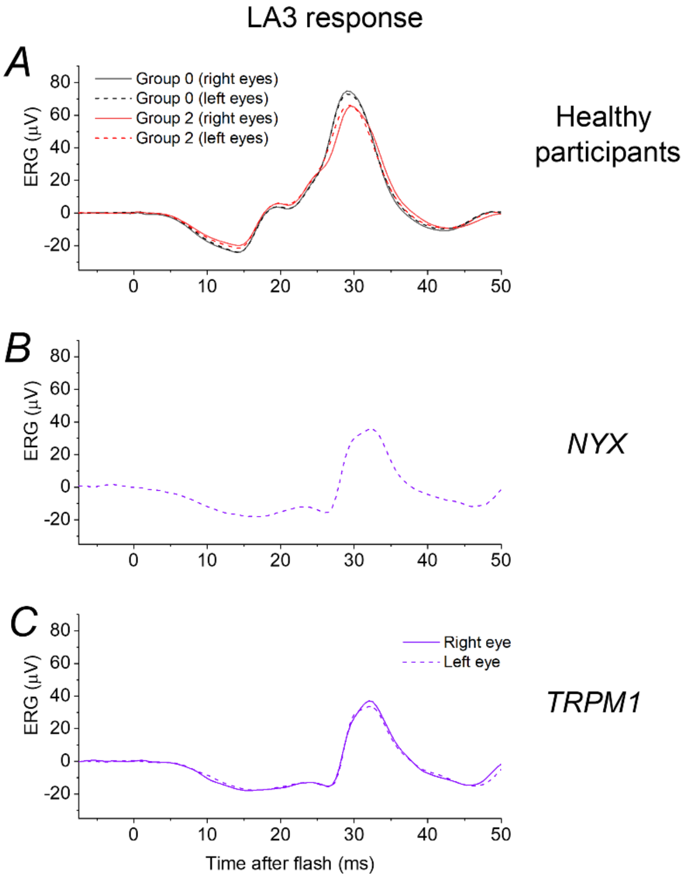

Figure S6. Response to international standard light-adapted flash from healthy participants and patients with loss of ON-pathway signals. *A,* Averaged light-adapted flash responses (LA 3 stimulus) for participants with no risk allele (Group 0, black traces) and those homozygous for the risk allele (Group 2, red traces). Solid and dashed traces show averages of right eye and left eye recordings respectively. Data are replotted from Figure 1*I*. *B*, Response to the same stimulus recorded from patient with *NYX*-associated complete congenital stationary night blindness (corresponding to purple trace in Supplementary Figure 5*A*). *C*, Response to the same stimulus recorded from patient with *TRPM1*-associated complete congenital stationary night blindness (corresponding to purple traces in Supplementary Figure 5*D*). The start of the b-wave in both patients occurs at a similar time to the time window over which the b-waves differ between Groups 0 and 2 in panel *A*. (The patients were myopic with long axial lengths, whilst the healthy subjects mostly had minimal refractive error, and so small differences in timing between healthy participants and the patients might be attributable to this.)

.

Fig. S7.

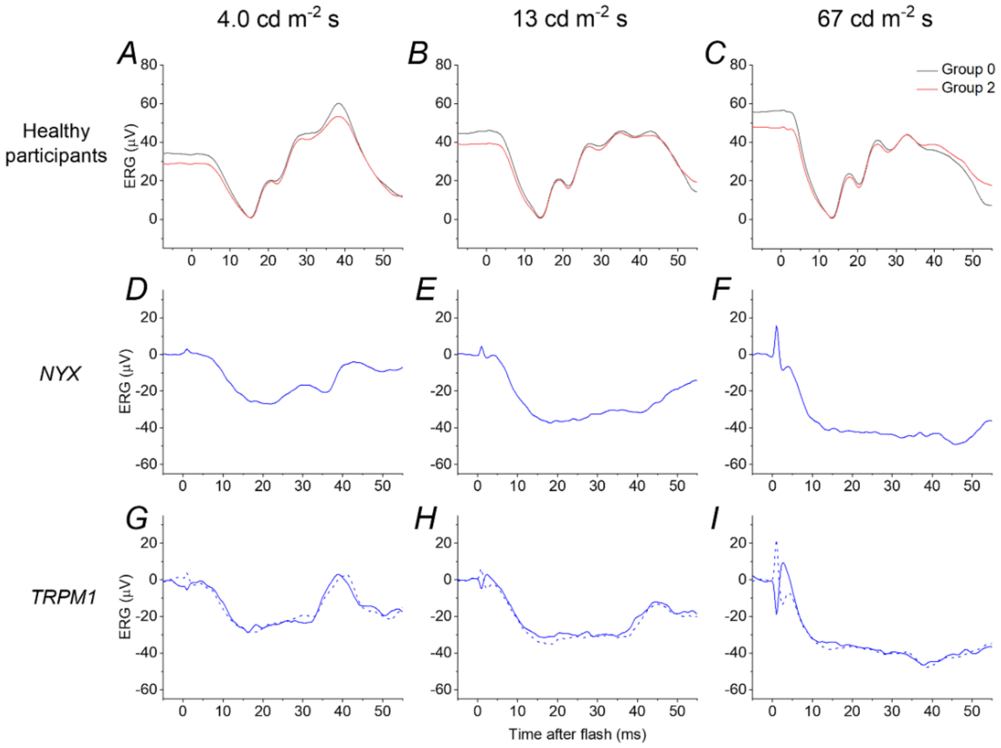

Figure S7. Cone-driven ERGs to flashes delivered in the presence of the dim rod-saturating blue background. *A-C*, The upper panels show the same ERGs depicted in Fig. 2 in the main text (middle panels), but the responses have been shifted on the y-axis by the respective a-wave amplitudes (so the a-wave trough is now at the baseline) to allow selective comparison of b-waves. B-wave amplitudes are similar for both groups, particularly for the brighter flashes. *D-F*, Responses to identical stimuli recorded from a patient with loss of ON-bipolar cell signals (the same ERGs depicted in the lower panels of Fig. 3 in the main text). These responses show that there is a small upward deflection in the dimmer flash response (panel *D*) at the time of the b-wave in the upper panel (panel *A*), but this is absent from the brighter flash responses. This is consistent with any difference in b-wave between the groups in the upper panels being attributable to OFF bipolar cell signals. *G-H*, Responses from another patient with loss of ON-bipolar cells signals (the same ERGs depicted in the lower panels of Supplementary Figure 5).

Table S1.

Table S1. Results from multivariate analysis of association between amplitudes of international standard ERGs and the genetic locus of interest. Beta denotes the effect size found in the model. *$p<0.05$

**ERG component**	**Beta**	**Standard error**	***p* value**
DA 0.01 b-wave	-0.079	0.051	0.121
DA 3 a-wave	-0.029	0.053	0.588
DA 3 b-wave	0.036	0.045	0.428
DA 10 a-wave	0.044	0.055	0.423
DA 10 b-wave	0.074	0.051	0.149
LA 30 Hz peak	0.100	0.050	0.046*
LA 3 a-wave	-0.102	0.051	0.046*
LA 3 b-wave	-0.066	0.043	0.129

Table S2.

Table S2. Results from multivariate analyses of association between amplitudes of experimentally derived rod and cone ERG parameters and the genetic locus of interest. A separate analysis was performed for cone-driven and rod-driven parameters as described in the Methods. Beta denotes the effect size found in the models. **p*<0.05

**Rod or cone-driven responses**	**Flash strength (cd m^{-2} s)**	**ERG component**	**Beta**	**Standard error**	***p* value**
Cone	0.67	a-wave	0.055	0.051	0.280
		b-wave	-0.071	0.049	0.148
	4.0	a-wave	-0.010	0.043	0.809
		b-wave	0.004	0.043	0.923
	13	a-wave	0.017	0.038	0.650
		b-wave	0.013	0.040	0.743
	67	a-wave	-0.126	0.052	0.015*
		b-wave	0.037	0.046	0.426
Rod	0.67	a-wave	-0.022	0.044	0.612
		b-wave	0.060	0.035	0.086
	4.0	a-wave	0.008	0.041	0.841
		b-wave	-0.026	0.027	0.348
	13	a-wave	0.005	0.040	0.895
		b-wave	-0.030	0.035	0.393
	67	a-wave	0.018	0.091	0.841
		b-wave	-0.102	0.099	0.303
